# Epidemiology of autoimmune liver disease in Korea: evidence from a nationwide real-world database

**DOI:** 10.1186/s13023-024-03086-0

**Published:** 2024-04-29

**Authors:** Jihye Lim, Hwa Jung Kim

**Affiliations:** 1 Division of Gastroenterology and Hepatology, Department of Internal Medicine, Yeouido St. Mary’s Hospital, College of Medicine, The Catholic University of Korea, Seoul, Republic of Korea; 2 Department of Clinical Epidemiology and Biostatistics Asan Medical Center, University of Ulsan College of Medicine, 88 Olympic-ro 43-gil, Songpa-gu, Seoul, 05505 Republic of Korea

**Keywords:** Epidemiology, Hepatitis, autoimmune, Liver cirrhosis, biliary, Cholangitis, sclerosing

## Abstract

**Background:**

Autoimmune hepatitis (AIH), primary biliary cholangitis (PBC), and primary sclerosing cholangitis (PSC) are all immune-mediated chronic inflammatory liver diseases. Autoimmune liver diseases are rare, making identification and treatment difficult. To improve clinical outcomes and enhance patient quality of life, we performed an epidemiological study of autoimmune liver diseases based on real-world comprehensive data.

**Results:**

We used National Health Insurance Service claims data in Korea from 2005 to 2019. Patients were identified using the International Classification of Disease 10th Revision code, and rare intractable disease codes assigned according to the strict diagnostic criteria. In the AIH cohort, 8,572 (83.9%) were females and the mean age at diagnosis was 56.3 ± 14.3 years. PBC also showed female dominance (83.3%) and the mean age was 57.8 ± 12.6 years. Patients with PSC showed no sex predominance and had a mean age of 57.8 ± 21.5 years. During the study period, there were 10,212, 6,784, and 888 AIH, PBC, and PSC patients, respectively. The prevalence of AIH, PBC, and PSC in 2019 were 18.4, 11.8, and 1.5 per 100,000 population, while the corresponding incidences were 2.3, 1.4, and 0.3 per 100,000 population, respectively. Analysis of sex-age-standardized data showed that the annual prevalence of these diseases is increasing. The 10-year survival rates were 89.8%, 74.9%, and 73.4% for AIH, PBC, and PSC, respectively.

**Conclusions:**

The number of patients with autoimmune liver disease in South Korea is increasing over time. Further research on autoimmune liver disease is needed to fulfill unmet clinical needs.

**Supplementary Information:**

The online version contains supplementary material available at 10.1186/s13023-024-03086-0.

## Background

Autoimmune liver diseases (AILD), such as autoimmune hepatitis (AIH), primary biliary cholangitis (PBC), and primary sclerosing cholangitis (PSC), are rare immunologic liver disease [1–6]. Diagnosis of AILD is difficult as its incidence is only 0.002% per year accounting for only 5% of liver diseases [1–7]. Early diagnosis and treatment of AILD is important but is often challenging in real-world practice. Most AILD patients present with non-specific symptoms such as fatigue, malaise, pruritis, or abdominal pain [1–6]. However, it is the reasons for severe liver complications like cirrhosis, hepatic dysfunction, and primary liver cancer. It often involves other major causing osteoporosis, diabetes, pancreatitis, vasculitis, thyroid disorders, rheumatologic disease, and inflammatory bowel disease [1, 5, 6, 8]. The 10-year survival rates of AILD are 50–90%, requiring regular hospital visits, lowering the quality of life, and generating psychological stress [1–6].

Due to the rarity and severity of AILD, it is classified as a rare intractable disease (RID) in Korea [9]. The National Health Insurance Service (NHIS) system has provided a universal health care service for 52 million Koreans since 1989 [10]. In terms of risk-sharing initiatives, the government has designated over 1,100 diseases characterized by an affected population of fewer than 20,000 individuals, necessitating consistent medical care, as RIDs. The NHIS has implemented a co-payment assistance policy for RID patients, providing support for 90% of their medical expenditures [11]. The unique health system in Korea allows for the collection of data that reflect the real-world situation, facilitating thorough evaluation of AILD with a large sample size followed over a long duration [12].

To better understand AILD, long-term, unbiased, nation-wide epidemiological studies are needed. Unfortunately, previous studies are rare and have limitations regarding completeness and accuracy, which prevent the establishment of a clear conclusion [6, 7, 13–25]. In this study, we aimed to identify the incidence and prevalence of AILD in Korea, further assessing their medical needs and mortality rates using the highly completed and qualified nationwide claims data.

## Methods

### Data source

We used NHIS claims data collected from 2005 to 2019. The NHIS contains data on all Korean healthcare utilization and demographic information [26]. Healthcare utilization information includes both in- and out-patient medical use, including diagnosis, treatment costs, procedures, and operations. It also collects data on medication, including drug codes, dose, prescription date, and duration. Demographics include sex, age, socioeconomic status, and place of residence.

### Definition of AILD patients

We used both the International Classification of Disease 10th Revision (ICD-10) and RID codes to guarantee the accuracy of data. The credibility of the RID system is bolstered by requiring physician-validated diagnoses that rely on biochemical, histological, and clinical evidence [9, 27]. For AIH, RID diagnosis depends on specific laboratory and histologic findings: (1) elevated levels of aminotransferase and immunoglobulin G; (2) presence of characteristic autoantibodies; and (3) defined histological abnormalities excluding evidence for other liver diseases [2, 28]. Similarly, for PBC, individuals meeting at least two of the following conditions may be classified as RID beneficiaries: (1) cholestasis with alkaline phosphatase elevation without evidence of other causes; (2) presence of antimitochondrial antibody or PBC-specific antinuclear antibody immunofluorescence (sp100 or gp210); and (3) characteristic pathology with chronic, nonsuppurative cholangitis [3, 29]. PSC patients are identified by (1) characteristic cholestatic biochemical liver function abnormalities, (2) typical bile duct changes with multifocal strictures and segmental dilatations on cholangiography, (3) or with histology compatible to PSC for the patients with normal cholangiography [30]. The disease definitions are compatible with the diagnosis outlined in international guidelines. AIH patients were defined as those with the codes K754 and V175, PBC as those with K743 and V174, and PSC as those with K830 and V262. Patients with incomplete data of age and sex were excluded.

### Statistical analysis

Patients were enrolled from the first date of their RID application, representing a definite diagnosis. Prevalence was calculated for each age and sex-specific spectrum by dividing the total number of alive patients by the total population. Incidence was calculated as the total number of newly diagnosed patients divided by the at-risk population, annually. Prevalence and incidence are expressed as the number of cases per 100,000 population. Sex-age standardized incidence ratio (SIR) and sex-age standardized prevalence ratio (SPR), based on the population of 2019, were conducted to eliminate the bias from the periodical differences in demographics. Economic status is represented as quantiles based on income, and the Medicare beneficiary is meant for people unable to maintain a living. Preference for a regional hospital, representing an imbalance in medical supply, was identified by agreement between the residential area and hospital location at the first request of the RID application. Patients were followed up until the date of death or the last date of claim data (August 31, 2021). The hospitalization rate was calculated as the number of patients who required hospitalization due to AILD divided by the number of alive patients in the corresponding year. The same calculation was performed for ER visits and ICU admissions. (Supplementary Table 1).

## Results

### Baseline characteristics of AILD patients

The patients’ baseline characteristics by sex are summarized in Table 1. Among the 10,212 AIH patients, 8,572 (83.9%) were females and the mean age was 56.3 ± 14.3 years. More than 65% of patients diagnosed with AIH were in their 40 to 60 s. Of the 6,784 PBC patients, 83.3% were females. PBC was most commonly diagnosed in patients in their 50 to 60 s, with a mean of 57.8 ± 12.6 years. The 888 patients with PSC showed no sex dominance and the mean age was 57.8 ± 21.5 years. Approximately half of AILD patients were classified in the 4th − 5th quantile of economic status and were predominantly residents of the capital area (Seoul Special City or Gyeonggi-do province). The time from the first date of the claim for each AILD (for AIH, PBC, and PSC) as a main or sub-diagnosis to the date of RID claim was 111 ± 381, 106 ± 367, 580 ± 955 days, respectively (data not shown).

**Table 1:** Demographics of autoimmune liver disease patients at the time of diagnosis

	Autoimmune hepatitis		Primary biliary cholangitis		Primary sclerosing cholangitis	
	Male (*n* = 1,640)	Female (*n* = 8,572)	Male (*n* = 1,135)	Female (*n* = 5,649)	Male (*n* = 449)	Female (*n* = 439)
***Age group, year***						
0–9	11 (0.7%)	11 (0.1%)	9 (0.8%)	12 (0.2%)	14 (3.1%)	21 (4.8%)
10–19	60 (3.7%)	77 (0.9%)	14 (1.2%)	17 (0.3%)	8 (1.8%)	10 (2.3%)
20–29	97 (5.9%)	248 (2.9%)	30 (2.6%)	27 (0.5%)	38 (8.5%)	17 (3.9%)
30–39	144 (8.8%)	612 (7.1%)	74 (6.5%)	293 (5.2%)	51 (11.4%)	22 (5%)
40–49	248 (15.1%)	1380 (16.1%)	172 (15.2%)	1001 (17.7%)	35 (7.8%)	49 (11.2%)
50–59	380 (23.2%)	2483 (29%)	279 (24.6%)	1796 (31.8%)	80 (17.8%)	69 (15.7%)
60–69	431 (26.3%)	2204 (25.7%)	324 (28.6%)	1509 (26.7%)	76 (16.9%)	87 (19.8%)
70–79	217 (13.2%)	1283 (15%)	177 (15.6%)	823 (14.6%)	84 (18.7%)	82 (18.7%)
80–89	51 (3.1%)	267 (3.1%)	55 (4.9%)	167 (3%)	60 (13.4%)	74 (16.9%)
90–99	1 (0.1%)	7 (0.1%)	1 (0.1%)	4 (0.1%)	3 (0.7%)	7 (1.6%)
***Economic status***						
Medicare	87 (5.3%)	419 (4.9%)	79 (7%)	255 (4.5%)	81 (18%)	92 (21%)
1 Quintile (lowest)	194 (11.8%)	1204 (14.1%)	124 (10.9%)	743 (13.2%)	43 (9.6%)	49 (11.2%)
2 Quintile	171 (10.4%)	1087 (12.7%)	117 (10.3%)	745 (13.2%)	43 (9.6%)	48 (10.9%)
3 Quintile	238 (14.5%)	1314 (15.3%)	166 (14.6%)	823 (14.6%)	40 (8.9%)	67 (15.3%)
4 Quintile	347 (21.2%)	1688 (19.7%)	245 (21.6%)	1151 (20.4%)	88 (19.6%)	69 (15.7%)
5 Quintile (highest)	569 (34.7%)	2700 (31.5%)	385 (33.9%)	1829 (32.4%)	143 (31.9%)	109 (24.8%)
Missing	34 (2.1%)	160 (1.9%)	19 (1.7%)	103 (1.8%)	11 (2.5%)	5 (1.1%)
***Residence***						
Seoul special city	409 (24.9%)	2079 (24.3%)	327 (28.8%)	1619 (28.7%)	81 (18%)	81 (18.5%)
Incheon^*^	83 (5.1%)	445 (5.2%)	47 (4.1%)	401 (7.1%)	24 (5.4%)	26 (5.9%)
Gyeonggi-do^¥^	369 (22.5%)	1969 (23%)	292 (25.7%)	1474 (26.1%)	97 (21.6%)	108 (24.6%)
Daejeon^*^, Sejong^*^	50 (3.1%)	266 (3.1%)	17 (1.5%)	175 (3.1%)	7 (1.6%)	15 (3.4%)
Chungcheong-do^¥^	110 (6.7%)	521 (6.1%)	91 (8%)	315 (5.6%)	24 (5.4%)	31 (7.1%)
Busan^*^, Daegu^*^, Ulsan^*^	269 (16.4%)	1469 (17.1%)	141 (12.4%)	706 (12.5%)	61 (13.6%)	51 (11.6%)
Gyeongsang-do^¥^	177 (10.8%)	838 (9.8%)	92 (8.1%)	403 (7.1%)	71 (15.8%)	61 (13.9%)
Gwangju^*^	27 (1.7%)	132 (1.5%)	17 (1.5%)	86 (1.5%)	17 (3.8%)	7 (1.6%)
Jeolla-do^¥^	85 (5.2%)	477 (5.6%)	74 (6.5%)	270 (4.8%)	46 (10.2%)	46 (10.5%)
Gangwon-do^¥^	44 (2.7%)	276 (3.2%)	25 (2.2%)	147 (2.6%)	15 (3.3%)	8 (1.8%)
Jeju-do^¥^	15 (0.9%)	95 (1.1%)	12 (1.1%)	50 (0.9%)	6 (1.3%)	5 (1.1%)
Missing	2 (0.1%)	5 (0.1%)	0 (0.0%)	3 (0.1%)	0 (0.0%)	0 (0.0%)

Values are expressed as frequency (percentage)

^*^Metropolitan city or special self-governing city

^¥^Province

### AILD prevalence and incidence by sex, age, and trend over time

Patients with AIH and PBC were registered as RID since 2005, and those with PSC since 2014. In total, 10,212, 6,784, and 888 patients were diagnosed and claimed for AIH, PBC, and PSC during the study period, respectively (Supplementary Fig. 1).

**Fig. 1:**
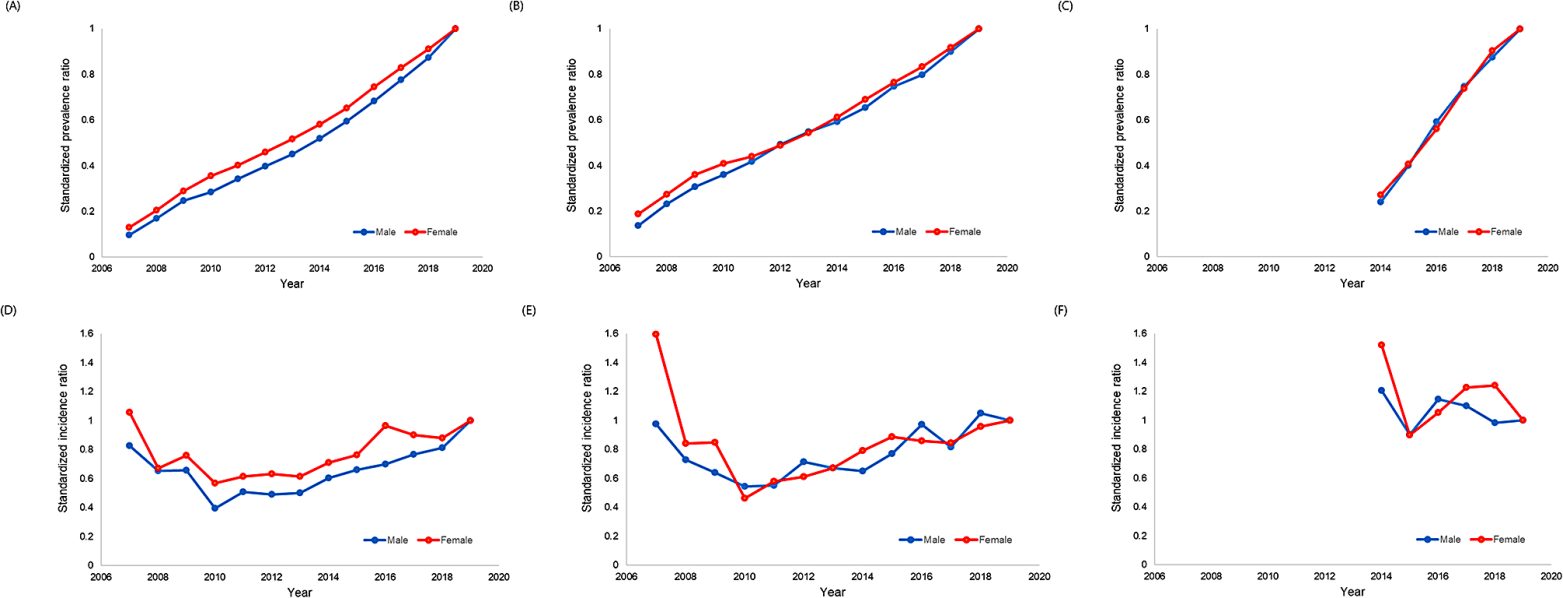
Sex-age-standardized prevalence of **(A)** AIH, **(B)** PBC, and **(C)** PSC, and the sex-age-standardized incidence of **(D)** AIH, **(E)** PBC, and **(F)** PSC. AIH, autoimmune hepatitis; PBC, primary biliary cholangitis; PSC, primary sclerosing cholangitis

The crude prevalence and incidence of AILD and tue number of AILD patients in 2019 are shown in Tables 2 and 3. The prevalence of AIH, PBC, and PSC was 18.4 (5.7 for males and 30.4 for females), 11.8 (3.7 for males and 19.8 for females), and 1.5 (1.5 for males and 1.5 for females) per 100,000 population, and the corresponding incidences were 2.3 (0.8 for males and 3.7 for females), 1.4 (0.5 for males and 2.3 for females), and 0.3 (0.3 for males and 0.3 for females) per 100,000 population, respectively, in 2019. The sex and age standardized prevalence and incidence ratios of AILD based on the population in 2019 are shown in Fig. 1. The sex-age-standardized annual prevalence and incidence of AIH, PBS, and PSC revealed an increase in the number of patients with SPR of 0.65, 0.69, and 0.40, and SIR of 0.74, 0.87, and 0.90 for AIH, PBC, and PSC in 2015, respectively.

**Table 2:** The number of autoimmune liver disease patients and crude prevalence by age group per 100,000 population in 2019

	Autoimmune hepatitis		Primary biliary cholangitis		Primary sclerosing cholangitis	
	Male (*n* = 1,450)	Female (*n* = 7,811)	Male (*n* = 957)	Female (*n* = 5,095)	Male (*n* = 379)	Female (*n* = 376)
0–9	10 (0.5)	10 (0.5)	9 (0.4)	12 (0.6)	14 (0.6)	17 (0.8)
10–19	58 (2.2)	76 (3.1)	13 (0.5)	16 (0.7)	8 (0.3)	10 (0.4)
20–29	96 (2.7)	237 (7.4)	27 (0.8)	26 (0.8)	37 (1)	17 (0.5)
30–39	138 (3.8)	589 (17.0)	72 [2]	278 [8]	45 (1.2)	21 (0.6)
40–49	234 (5.5)	1323 (32.3)	156 (3.7)	956 (23.3)	32 (0.8)	47 (1.1)
50–59	344 (8.0)	2343 (55.3)	247 (5.8)	1696 (40)	71 (1.7)	65 (1.5)
60–69	375 (12.7)	1987 (64.3)	276 (9.3)	1348 (43.6)	65 (2.2)	77 (2.5)
70–79	165 (10.6)	1057 (54.3)	124 [8]	639 (32.8)	62 [4]	66 (3.4)
80–89	30 (5.6)	185 (18.1)	32 [6]	120 (11.7)	44 (8.3)	51 [5]
90–99	0 (0.0)	4 (2.5)	1 (2.2)	4 (2.5)	1 (2.2)	5 (3.1)

Variables are shown as frequency (frequency per 100,000 population)

**Table 3:** The number of newly diagnosed autoimmune liver disease patients and crude incidence by age group per 100,000 population in 2019

	Autoimmune hepatitis		Primary biliary cholangitis		Primary sclerosing cholangitis	
	Male (*n* = 217)	Female (*n* = 952)	Male (*n* = 132)	Female (*n* = 592)	Male (*n* = 76)	Female (*n* = 67)
0–9	1 (0.0)	0 (0.0)	1 (0.0)	1 (0.0)	0 (0.0)	1 (0.0)
10–19	7 (0.3)	6 (0.2)	1 (0.0)	1 (0.0)	1 (0.0)	2 (0.1)
20–29	10 (0.3)	26 (0.8)	4 (0.1)	3 (0.1)	5 (0.1)	3 (0.1)
30–39	14 (0.4)	50 (1.4)	8 (0.2)	21 (0.6)	11 (0.3)	2 (0.1)
40–49	31 (0.7)	129 (3.1)	15 (0.4)	81 (2.0)	5 (0.1)	5 (0.1)
50–59	53 (1.2)	243 (5.7)	32 (0.7)	169 (4.0)	11 (0.3)	10 (0.2)
60–69	64 (2.2)	273 (8.8)	38 (1.3)	189 (6.1)	17 (0.6)	18 (0.6)
70–79	26 (1.7)	179 (9.2)	28 (1.8)	97 (5.0)	10 (0.6)	11 (0.6)
80–89	11 (2.1)	45 (4.4)	5 (0.9)	29 (2.8)	16 (3.0)	13 (1.3)
90–99	0 (0.0)	1 (0.6)	0 (0.0)	1 (0.6)	0 (0.0)	2 (1.2)

Variables are shown as frequency (frequency per 100,000 population)

### Medical facility use of AILD patients

We identified a regional difference in the use of medical facility for AILD diagnosis. Most patients who lived in capital, Seoul Special City (94.6%), used regional hospitals, followed by those who lived in the metropolitan city or special self-governing city (Busan, Daegu, Ulsan province [86.6%], and Jeju-do province [77.6%]). Conversely, most inhabitants of rural area (Chungcheong-do [67.7%] and Gyeongsang-do [66.7%]) visited hospitals in different districts. The diagnosis of AILD was mostly made and claimed for RID from internal medicine consultants (94.2%) consistent with 95.7% and 94.9% of AIH and PBC patients. In the case of PSC, general surgery department was responsible for 9.3% of diagnosis while internal medicine department was responsible for 72.3% of diagnosis (Supplementary Table 2).

**Fig. 2:**
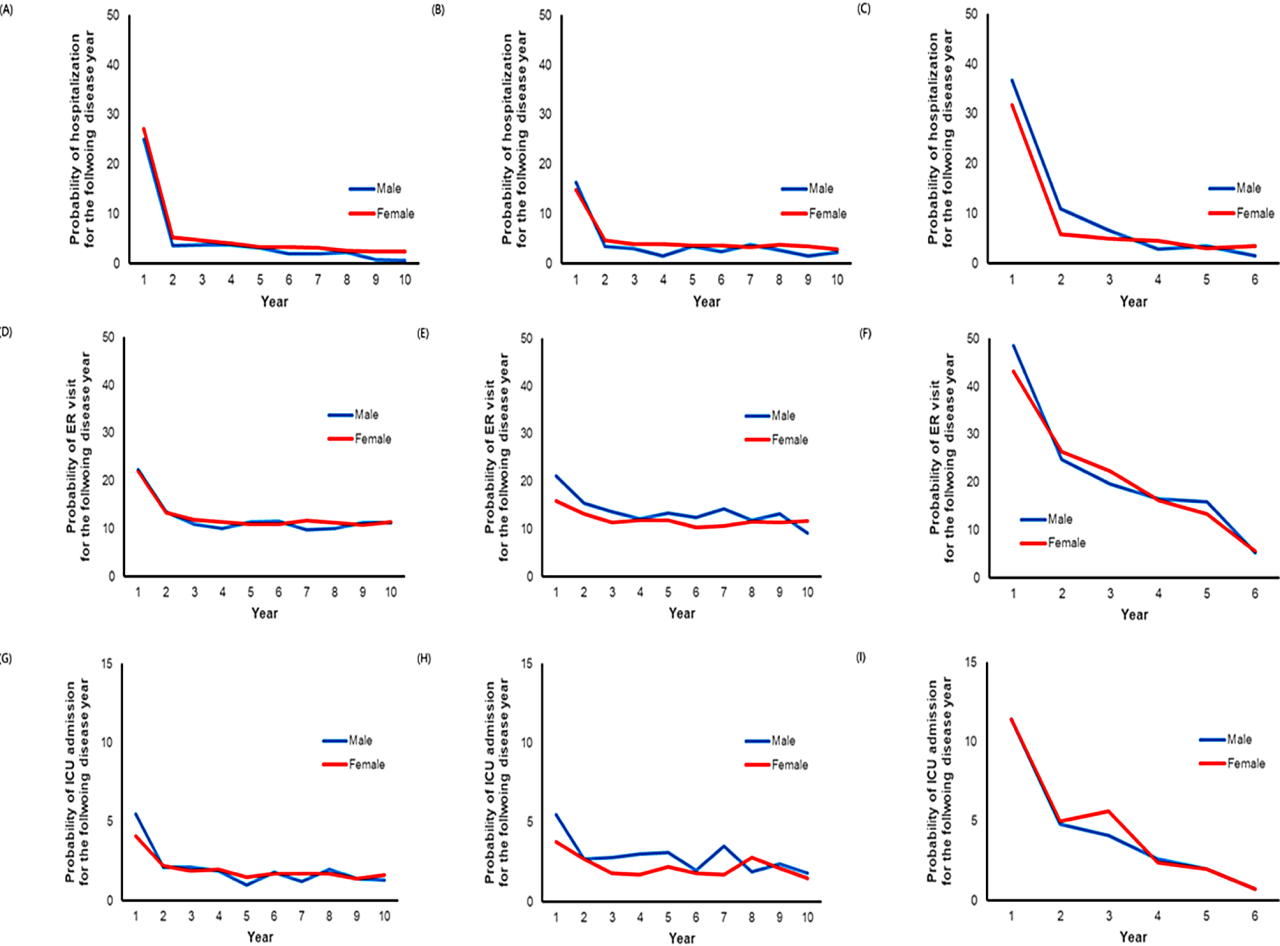
The probability of patients with AIH for **(A)** hospitalization, **(B)** ER visit, and **(C)** ICU admission after the diagnosis. Likewise, the probabilities for patients with PBC include **(D)** hospitalization, **(E)** ER visit, and **(F)** ICU admission after the diagnosis. Additionally, the probabilities for patients with PSC encompass **(G)** hospitalization, **(H)** ER visit, and **(I)** ICU admission after the diagnosis. AIH, autoimmune hepatitis; ER, emergency room; ICU, intensive care unit; PBC, primary biliary cholangitis; PSC, primary sclerosing cholangitis

**Fig. 3:**
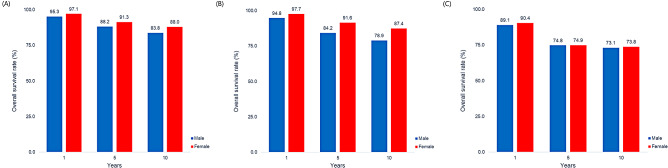
The 1-, 5-, and 10-year overall survival rates of **(A)** AIH, **(B)** PBC, and **(C)** PSC. AIH, autoimmune hepatitis; PBC, primary biliary cholangitis; PSC, primary sclerosing cholangitis

The follow-up durations of AIH, PBC, and PSC were 88.4 ± 52.1, 89.7 ± 52.4, and 50.1 ± 27.3, respectively. During the study period, hospitalization, ER visits, and ICU admissions were most frequent during the first year after diagnosis. In total, 26.9%, 15.1%, and 34.3% of AIH, PBC, and PSC patients were admitted to the hospital in the first year, respectively. Overall, 22.1%, 16.8%, and 45.9% of AIH, PBC, and PSC patients visited the ER, and 4.3%, 4.1%, and 11.4% required ICU care in the first year of diagnosis, respectively (Fig. 2). The 1-year, 5-year, and 10-year survival rates of each AILD were 99.0%, 97.2%, and 89.8%; 90.8%, 90.4%, and 74.9%; and 89.8%, 74.9%, and 73.4% for AIH, PBC, and PSC, respectively (Fig. 3).

## Discussion

In this study, we used nationwide claim data collected over 15 years to investigate the epidemiological characteristics of AILD. The prevalence of AIH, PBC, and PSC were 18.4, 11.8, and 1.5 per 100,000 population, while the corresponding incidence was 2.3, 1.4, and 0.3 per 100,000 population, respectively, in 2019. The 10-year survival rates were 89.8%, 74.9%, and 73.4%, respectively. This result reflects the clinical significance of AILD in Korea.

In our study, more than half of AILD patients are diagnosed in middle to late age. Most AIH and PBC patients were diagnosed in their fifties or sixties, while the age at diagnosis for PSC was spread more evenly between the age range of 50-80 s. AIH and PBC showed female dominancy (84.0% and 83.3% female patients, respectively), while PSC was not gender dependent (49.4% female patients). The age at the initial diagnosis and female predominance of AIH (76–80% of females with a median age of 43–68 years) and PBC (54-86.5% of females with a mean age of 52.8–64.7 years) are similar to the findings of other studies [18, 20–23]. However, the demographics of the PSC population differed from previous studies, which generally showed that PSC was diagnosed in middle age (median age of 35–55 years) with male predominancy (55–75%) [22–25]. This difference might be attributed to the epidemiological variations in the disease entity or the higher probability of detecting laboratory abnormalities in individuals aged 20 years or older who undergo national healthcare checkups every two years [31].

Previous epidemiology studies about AILD are few and incomplete with considerably different results. The prevalence of AIH ranges from 4.0 to 42.9 per 100,000 population, and the incidence ranges from 0.5 to 3.0 per 100,000 population per year [13–17]. Those of PBC were 10.0 to 58.1 and 0.78 to 5.23 per 100,000 population per year, respectively [18–21]. Likewise, the prevalence and incidence of PSC ranged from 1 to 16.2 and 0.04 to 1.3 per 100,000 population per year, respectively [6, 22–25]. These discrepancy are based on the study limitations; for example, some were only hospital or community-based and used ambiguous disease definitions [7]. Epidemiological studies of rare diseases should be population-based to avoid non-random selection bias. The diagnostic criteria must adhere to international disease definition based on clinical results. The results must be standardized to eliminate innate bias from regional and demographical differences. With these considerations in mind, our results reflect unbiased and reproductive real-world data on AILD with comparability to other studies.

Our results show that the number of newly diagnosed AILD patients in Korea has been increasing since 2010, as has the sex-age-standardized incidence of AIH and PBC. The total number of patients newly diagnosed with AIH was 507 in 2010 and 1,169 in 2019. In the case of PBC, the numbers were 271 and 724 for the respective years.

The sudden increase in the number of AILD patients in the early days may have been influenced by the health and welfare policy. AILD patients who were previously diagnosed and treated would be enrolled as RID patients in the early days of the RID policy, which began at once as is true for PSC in 2014. The prevalence of AILD has also increased rapidly over time. The cumulative number of AILD patients has been rising.

The AILD diagnosis and care is challenging [1, 3, 4, 6]. In our study, the duration of AILD confirmation (initial KCD code to RID code date for AILD) took about 3.7 to 20.7 months. AILD diagnosis needs to exclude other liver diseases caused by viral, drug, or hereditary diseases, and, sometimes, requires pathologic confirmation. The demand for medical facility use was mostly focused on the first year of diagnosis and decreased from the second year. Previous studies have shown that the 10-year survival rates of AIH, PBC, and PSC were 82–91%, 38–94%, and 64–92%, respectively [18, 32–35]. In our study population, the 10-year survival rates of AIH, PBC, and PSC were 89.8%, 74.9%, and 73.4%, respectively.

The strength of this study is the use of a nationwide cohort with almost 100% coverage of the Korean population over a long period of time. However, there are some limitations. First, due to the inherent limitations of claims data, we could not retrieve medical records, making it impossible to assess the clinical finding such as laboratory, image, or histological results. Thanks to RID system, our data is accurate and reliable overcoming potential confounding biases. The sensitivity and specificity of ICD-10 code with RID code in other rare disease were 92–98% [36]. Also, we applied the same diagnostic criteria for AIH, PBC and PSC, it reveals the trend of AILD. In this study, we mainly focused on the epidemiology of AILD and did not collect other medical conditions that might affect AILD. We plan to further investigate comorbidities, liver functions, or malignancies which might affect clinical course of AILD. Lastly, we were not able to reveal the reasons of medical use in this study. The hospital utilization were most frequent in the first year of diagnosis. Patients with AILD might either present with the early stage of the disease without significant liver dysfunction for some period under proper management, or with the end stage of liver disease requiring ER visit or ICU admission. More specific data regarding the reasons for medical utilization in the long term is required to understand better about the clinical presentation of AILD.

In conclusion, this nationwide study demonstrated the epidemiology of AILD in Korea. This information will facilitate the understanding of AILD using a real-world database. As the incidence and prevalence of AILD increases, further studies to improve the overall well-being of AILD patients are warranted.

### Electronic supplementary material

Below is the link to the electronic supplementary material.

Supplementary Material 1

## Data Availability

The data of this article is provided by the Korea NHISS. However, restrictions apply to the availability of these data, which were used under license for the current study, and so are not publicly available.

## Abbreviations

AIH: Autoimmune hepatitis
AILD: Autoimmune liver disease
ICD-10: International Classification of Disease 10th Revision
NHIS: National Health Insurance Service
PBC: Primary biliary cholangitis
PSC: Primary sclerosing cholangitis
RID: Rare intractable disease
SIR: Sex-age standardized incidence rate
