# Supplementary material for: Epidemiology of autoimmune liver disease in Korea: evidence from a nationwide real-world database
Source: Orphanet J Rare Dis. 2024 Apr 29;19:178. doi: 10.1186/s13023-024-03086-0 (PMC11057181; doi:10.1186/s13023-024-03086-0)
Supplement: Supplementary file 1 — Supplementary Material 1 [file 13023_2024_3086_MOESM1_ESM.docx]

**Supplementary materials**

**Epidemiology of autoimmune liver disease in Korea: Evidence from a nationwide real-world database**

**Jihye Lim, Hwa Jung Kim**

**Supplementary Figure 1**

**Supplementary Table 1**

**Supplementary Table 2**

**Supplementary Figure 1.** Flowchart of a study group of (A) AIH, (B) PBC, and (C) PSC

| **(A)** | Patients claimed AIH (ICD 10; K754) as the main or sub diagnosis  between January 2005 and December 2019 | | |
| --- | --- | --- | --- |
|  | (n=14,673) | | |
|  |  |  | |
|  | Patients registered in the RID system (V175) and got an application for co-payment assistance | | |
|  | (n=10,236) | | |
|  |  |  | |
|  |  |  | Exclusion |
|  |  |  | Patients with incomplete demographic data (n=24) |
|  |  |  | |
|  | Study population of AIH | | |
|  | (n=10,212) | | |
|  |  |  |  |
|  |  |  |  |
| **(B)** | Patients claimed PBC (ICD 10; K743) as the main or sub diagnosis  between January 2005 and December 2019 | | |
|  | (n=8,767) | | |
|  |  |  | |
|  | Patients registered in the RID system (V174) and got an application for co-payment assistance | | |
|  | (n=6,804) | | |
|  |  |  | |
|  |  |  | Exclusion |
|  |  |  | Patients with incomplete demographic data (n=20) |
|  |  |  | |
|  | Study population of PBC | | |
|  | (n=6,784) | | |
|  |  |  |  |
|  |  |  |  |
| **(C)** | Patients claimed PSC (ICD 10; K830) as the main or sub diagnosis  between January 2005 and December 2019 | | |
|  | (n=11,910) | | |
|  |  |  | |
|  | Patients registered in the RID system (V262) and got an application for co-payment assistance | | |
|  | (n=889) | | |
|  |  |  | |
|  |  |  | Exclusion |
|  |  |  | Patients with incomplete demographic data (n=1) |
|  |  |  | |
|  | Study population of PSC | | |
|  | (n=888) | | |

AIH, autoimmune hepatitis; PBC, primary biliary cholangitis; PSC, primary sclerosing cholangitis; ICD 10, International Classification of Disease 10th Revision; RID, rare intractable disease

**Supplementary Table 1.** The definition of outcome variables

| **Incidence =** | Number of new AILD cases of disease over a specific year | x 100,000 |
| --- | --- | --- |
|  | Average population at risk |  |
|  |  |  |
| **Prevalence =** | Number of current AILD cases over a specific year | x 100,000 |
|  | Average population |  |
|  |  |  |
| **Admission rate =** | Number of patients hospitalized for AILD during a specific year | x 100 |
|  | Number of alive patients in a specific year |  |
|  |  |  |
| **ER visit rate =** | Number of patients visited ER for AILD during a specific year | x 100 |
|  | Number of alive patients in a specific year |  |
|  |  |  |
| **ICU admission rate =** | Number of patients admitted to ICU for AILD during a specific year | x 100 |
|  | Number of alive patients in a specific year |  |
|  |  |  |

AILD; autoimmune liver disease; ER, emergency room; ICD, intensive care unit

**Supplementary Table 2.** Utilization of medical institutions in residential areas of AILD patients

|  | Autoimmune hepatitis | | Primary biliary cholangitis | | | Primary sclerosing cholangitis | | |
| --- | --- | --- | --- | --- | --- | --- | --- | --- |
|  | Male  (n=1640) | Female  (n=8572) | | Male  (n=1135) | Female  (n=5649) | | Male  (n=449) | Female  (n=439) |
| Seoul Special City | 391 (95.6%) | 1967 (94.6%) | | 314 (96%) | 1530 (94.5%) | | 75 (92.6%) | 71 (87.7%) |
| Incheon^*^ | 57 (68.7%) | 283 (63.6%) | | 30 (63.8%) | 275 (68.6%) | | 12 (50%) | 17 (65.4%) |
| Gyeonggi-do^¥^ | 179 (48.5%) | 1124 (57.1%) | | 159 (54.5%) | 854 (57.9%) | | 43 (44.3%) | 51 (47.2%) |
| Daejeon^*^, Sejong^*^ | 36 (72%) | 198 (74.4%) | | 11 (64.7%) | 123 (70.3%) | | 3 (42.9%) | 7 (46.7%) |
| Chungcheong-do^¥^ | 37 (33.6%) | 147 (28.2%) | | 37 (40.7%) | 101 (32.1%) | | 11 (45.8%) | 20 (64.5%) |
| Busan^*^, Daegu^*^, Ulsan^*^ | 227 (84.4%) | 1275 (86.8%) | | 112 (79.4%) | 554 (78.5%) | | 48 (78.7%) | 39 (76.5%) |
| Gyeongsang-do^¥^ | 67 (37.9%) | 242 (28.9%) | | 37 (40.2%) | 111 (27.5%) | | 44 (62%) | 46 (75.4%) |
| Gwangju^*^ | 17 (63%) | 96 (72.7%) | | 12 (70.6%) | 47 (54.7%) | | 9 (52.9%) | 3 (42.9%) |
| Jeolla-do^¥^ | 38 (44.7%) | 237 (49.7%) | | 43 (58.1%) | 110 (40.7%) | | 34 (73.9%) | 32 (69.6%) |
| Gangwon-do^¥^ | 25 (56.8%) | 152 (55.1%) | | 13 (52%) | 47 (32%) | | 8 (53.3%) | 6 (75%) |
| Jeju-do^¥^ | 11 (73.3%) | 79 (83.2%) | | 10 (83.3%) | 35 (70%) | | 4 (66.7%) | 3 (60%) |
| Missing | 0 (0.0%) | 0 (0.0%) | | 0 (0.0%) | 0 (0.0%) | | 0 (0.0%) | 0 (0.0%) |

Values are expressed as frequency (percentage).

^*^Metropolitan city or special self-governing city

^¥^Province
